# Beyond Modified Transudates: Diagnostic Performance of the Feline Infectious Peritonitis Effusion Index on the Sysmex XN-1000V

**DOI:** 10.3390/pathogens15101042

**Published:** 2026-10-02

**Authors:** Ricardo Lopes, Filipe Sampaio, Hugo Lima de Carvalho, Cristina Costa Santos, Melanie Ginder, Cátia Fernandes, Gonçalo Barros, Luís Cardoso, Elsa Leclerc Duarte, Alessia Giordano, Ana Cláudia Coelho

**Affiliations:** 1 CEDIVET Veterinary Laboratories, Lionesa Business Hub, R. Lionesa 446 C24, 4465-671 Leça do Balio, Portugal; hugo.carvalho@cedivet.pt (H.L.d.C.); goncalo.barros@cedivet.pt (G.B.); 2 Department of Veterinary Sciences, University of Trás-os-Montes e Alto Douro (UTAD), 5000-801 Vila Real, Portugal; lcardoso@utad.pt (L.C.); accoelho@utad.pt (A.C.C.); 3 Department of Veterinary and Animal Sciences, CESPU University, 4585-116 Gandra, Portugal; 4 UNIPRO—Oral Pathology and Rehabilitation Research Unit, CESPU University, 4585-116 Gandra, Portugal; 5 Cytology and Hematology Diagnostic Services, Laboratory of Histology and Embryology, Department of Microscopy, ICBAS-School of Medicine and Biomedical Sciences, University of Porto (U. Porto), Rua de Jorge Viterbo Ferreira, 228, 4050-313 Porto, Portugal; 6 RISE-Health, Department of Community Medicine, Information and Health Decision Sciences, Faculty of Medicine, University of Porto (U. Porto), Alameda Prof. Hernâni Monteiro, 4200-319 Porto, Portugal; csantos@med.up.pt; 7 Sysmex Europe SE, 22297 Hamburg, Germany; ginder.melanie@sysmex-europe.com; 8 AniCura Santa Marinha Veterinary Hospital, R. Dom Henrique de Cernache 183, 4400-625 Vila Nova de Gaia, Portugal; catia.fernandes@anicura.pt; 9 Animal and Veterinary Research Centre (CECAV), Associate Laboratory for Animal and Veterinary Sciences (AL4AnimalS), University of Trás-os-Montes e Alto Douro (UTAD), 5000-801 Vila Real, Portugal; 10 Department of Veterinary Medicine, School of Science and Technology, University of Évora, Polo da Mitra, Apartado 94, 7002-554 Évora, Portugal; emld@uevora.pt; 11 Mediterranean Institute for Agriculture, Environment and Development (MED), Global Change and Sustainability Institute (CHANGE), University of Évora, Polo da Mitra, Apartado 94, 7002-554 Évora, Portugal; 12 Department of Veterinary Medicine and Animal Sciences, University of Milan, 26900 Lodi, Italy; alessia.giordano@unimi.it

**Keywords:** clinical pathology, coronavirus, delta total nucleated cells, diagnostic accuracy, effusions, feline infectious peritonitis, FIP Effusion Index, flow cytometry, Sysmex XN-1000V

## Abstract

The antemortem clinical and clinicopathological diagnosis of feline infectious peritonitis (FIP) remains challenging, particularly when effusions deviate from the classically protein-rich, low-to-moderate-cellularity phenotype. The FIP Effusion Index, defined as the ratio of delta total nucleated cells (ΔTNC) to the albumin-to-globulin (A/G) ratio, was developed and initially evaluated in 50 effusions. Subsequent European Advisory Board on Cat Diseases (ABCD) guidelines recommended evaluation in larger cohorts and in cats with confirmed FIP. This study retrospectively evaluated the prespecified threshold (≥7.54) in 176 independent feline effusion samples analysed on the Sysmex XN-1000V and classified by effusion FCoV RT-qPCR, without independent confirmation of FIP status. Relative to this molecular reference, the FIP Effusion Index yielded a sensitivity of 97.8% (95% confidence interval [CI], 92.4–99.7%), a specificity of 88.1% (95% CI, 79.2–94.1%) and an area under the receiver operating characteristic curve (AUC) of 0.962 (95% CI, 0.928–0.995). Discrimination exceeded that of ΔTNC alone (AUC, 0.947; ΔAUC, 0.014; *p* = 0.012) and A/G alone (AUC, 0.887; ΔAUC, 0.074; *p* = 0.012). In exudates, sensitivity remained 100% (95% CI, 80.5–100%) and specificity was 82.8% (95% CI, 64.2–94.2%). Among reference-negative effusions, false-positive classification was more frequent in septic than in non-septic samples (6/19 vs. 4/65; odds ratio, 7.04; 95% CI, 1.74–28.53; *p* < 0.01). These findings demonstrate that the prespecified FIP Effusion Index threshold retained very high sensitivity and strong discrimination for molecular-reference positivity across a broader spectrum of feline effusions. Future studies should evaluate the FIP Effusion Index within a sequential in vivo diagnostic algorithm, using negative results to support rule-out and positive results to prompt confirmation by FCoV antigen detection using immunocytochemistry and/or immunohistochemistry.

## 1. Introduction

Feline infectious peritonitis (FIP) is a systemic inflammatory disease that develops in a minority of cats infected with feline coronavirus (FCoV) and remains one of the most challenging diagnoses in feline medicine [1,2,3]. The availability of effective antiviral therapy has further increased the clinical importance of establishing an early and reliable diagnosis to permit prompt treatment [4,5,6,7,8,9]. When an effusion is present, its analysis should be prioritised, as the combination of cytology, biochemical assessment and direct detection of FCoV RNA or antigen provides substantially greater diagnostic information than peripheral blood testing [1,10,11,12]. Characteristic histopathological lesions together with intralesional FCoV antigen detection by immunohistochemistry (IHC) constitute the reference standard for definitive FIP diagnosis, although tissue sampling is invasive and may be impractical in clinically ill cats [1,2,10,11,12]. FCoV reverse-transcription quantitative polymerase chain reaction (RT-qPCR) on effusion samples is rapidly available and has shown high sensitivity and specificity, often reaching 100% in several studies [2,13,14,15,16,17,18,19,20,21]. Longstaff et al. [20] reported 85% sensitivity and 100% specificity in effusions, while Doenges et al. [21] reported 88.9% sensitivity and 100% specificity in cell-free body-cavity effusions, relative to disease classifications that included FCoV antigen detection by IHC. Although histopathology with intralesional FCoV antigen detection remains the reference standard, effusion RT-qPCR was selected as the molecular reference classification for this study because effusion sampling is less invasive than tissue biopsy, results are rapidly available and the assay is suitable for routine diagnostic workflows.

Classically, FIP-associated effusions are usually protein-rich, yellow to straw-coloured, viscous and sticky, often with fibrin or clot formation and frequently yielding a modified-transudative phenotype [3,22,23,24]. Cytologically, they usually contain a mixed pyogranulomatous population of non-degenerate neutrophils, macrophages or monocytes and few lymphocytes in a proteinaceous granular background [3,22,25,26,27]. Nevertheless, the biological spectrum is wider than this traditional description implies. Current FIP diagnostic guidelines recognise that total nucleated cell counts (TNCCs) can be substantially increased in FIP, including values exceeding 20 × 10^9^/L, and that secondary bacterial peritonitis may coexist with FIP [1,2,3,28,29]. From a diagnostic perspective, this means that effusion phenotype should be interpreted cautiously: an exudative pattern does not exclude FIP, and severe inflammatory conditions unrelated to FIP may share some biochemical features commonly considered supportive of the disease [1,22,25,28].

Among inexpensive effusion-based diagnostic tests, the albumin-to-globulin (A/G) ratio and Rivalta test remain widely used [2,3,30]. Both, however, have important limitations. A low A/G ratio reflects the inflammatory protein profile of FIP but is not disease-specific, whereas a positive Rivalta reaction may occur in septic peritonitis and lymphoma-related effusions [2,17,31,32,33]. Diagnostic guidelines place a low effusion A/G ratio in the “more likely” rather than the “definitive” category, with values <0.4 increasing suspicion and >0.8 making FIP less likely [2,3,30,32]. The discordance between cellularity measurements obtained from the white blood cell differential (WDF) and white cell nucleated (WNR) analytical channels of the Sysmex XN-1000V provides a further diagnostic signal [1,11]. The parameter termed delta total nucleated cells (ΔTNC) has shown high diagnostic accuracy in feline effusions analysed on Sysmex haematology platforms [34,35].

The FIP Effusion Index was introduced in 2024 by our research group to integrate this cellular-channel phenomenon with the biochemical information contained in the A/G ratio [1]. In the derivation cohort of 50 effusions, a FIP Effusion Index threshold of ≥7.542 yielded 96.3% sensitivity and 100% specificity relative to effusion FCoV RT-qPCR status [1]. Importantly, that initial investigation excluded septic, unclear and inconclusive cytological samples, thereby evaluating a comparatively restricted diagnostic spectrum. The detailed 2026 European Advisory Board on Cat Diseases (ABCD) FIP guidelines subsequently highlighted the FIP Effusion Index as promising, specifically noting the need for evaluation in a larger number of samples and in more firmly confirmed FIP cases before routine recommendation [30].

The principal challenge in validating a diagnostic index is not merely to reproduce its performance in a population resembling the derivation cohort, but to determine whether a prespecified threshold remains informative when confronted with the heterogeneous inflammatory and neoplastic conditions encountered in routine practice. Re-optimising the threshold in each new dataset would inflate apparent performance and would not constitute a stringent validation. Accordingly, the present study retained ≥7.54 as the primary, prespecified clinical threshold and treated any data-driven threshold analysis as exploratory.

The primary objective was to assess the diagnostic performance of the FIP Effusion Index in a substantially larger, expanded-spectrum cohort of feline effusions using effusion FCoV RT-qPCR status as the molecular reference classification. Secondary objectives were to: (i) compare the continuous discrimination of the FIP Effusion Index with its individual components, ΔTNC and A/G; (ii) compare the prespecified FIP Effusion Index threshold with previously published thresholds for ΔTNC, A/G and the Rivalta test; (iii) determine whether sensitivity was retained in exudative effusions, which extend beyond the classically modified-transudative phenotype and may present a greater risk of misclassification; and (iv) investigate septic inflammation as a source of false-positive results. We hypothesised that the FIP Effusion Index would retain high sensitivity across effusion phenotypes, while severe septic inflammation would represent a biologically plausible limitation to specificity.

## 2. Materials and Methods

### 2.1. Study Design, Setting and Diagnostic Records

This retrospective, expanded-spectrum diagnostic-accuracy study was conducted using feline effusion samples submitted for routine diagnostic investigation to CEDIVET Veterinary Laboratories (Porto, Portugal) between 1 March 2025 and 1 March 2026. Eligible samples included effusions across different cytological and biochemical phenotypes, including modified transudates, exudates and septic inflammatory effusions. All performance estimates were calculated relative to effusion FCoV RT-qPCR classification and not to independently confirmed FIP status.

Effusion records were eligible for inclusion when a valid FCoV RT-qPCR result was available together with all laboratory measurements required to calculate the FIP Effusion Index, namely total nucleated cell counts from the WDF (TNCC-WDF) and WNR (TNCC-WNR) channels and the effusion A/G ratio. TNCC-WDF and TNCC-WNR measurements were obtained using the Sysmex XN-1000V analytical channels with Lysercell WDF (Cat. No. CV377552), Fluorocell WDF (Cat. No. AA325279), Lysercell WNR (Cat. No. DWX-50102) and Fluorocell WNR (Cat. No. M375584) reagents, respectively (Sysmex Corporation, Kobe, Japan). A Rivalta test result, effusion phenotype and septic classification were additionally required for the corresponding comparative and subgroup analyses.

Samples were submitted in ethylenediaminetetraacetic acid (EDTA; 4 mL; Cat. No. 454209, Greiner Bio-One, Kremsmünster, Austria) and serum tubes (4 mL; Cat. No. 454092, Greiner Bio-One, Kremsmünster, Austria) and processed within 18–24 h of collection, in accordance with the analytical protocol used in the original study [1]. Records were excluded when the molecular result was analytically invalid, the available sample volume was insufficient to complete the required analyses, or any measurement necessary to derive the primary index test, namely TNCC-WDF, TNCC-WNR, or the effusion A/G ratio, was unavailable. Specifically, samples with invalid amplification controls, insufficient volume, or missing TNCC-WDF, TNCC-WNR, or A/G ratio values were excluded. RT-qPCR results were interpreted according to the manufacturer’s qualitative criteria: samples with valid amplification controls and target amplification within the analytical run were classified as molecular-reference positive, whereas valid samples without target amplification were classified as molecular-reference negative. No separate borderline or indeterminate molecular category was defined for this study, and analytically invalid results were excluded from diagnostic-accuracy analyses. No records meeting the initial eligibility criteria were excluded for these reasons. Unlike the original derivation study, septic effusions were intentionally retained to evaluate the FIP Effusion Index under a broader spectrum of inflammatory conditions encountered in routine diagnostic practice.

The statistical unit was the individual effusion sample. All 176 samples represented independent observations from 176 individual cats, with no cat contributing more than one sample to the primary analysis. All eligible samples fulfilling the inclusion criteria during the predefined study period were included, and no imputation was performed for variables used in the primary diagnostic analyses. The study was reported according to the Standards for Reporting of Diagnostic Accuracy (STARD) [36].

### 2.2. Laboratory Analyses and Effusion Classification

Cytological examination was performed on direct smears, squash preparations or cytocentrifuged specimens, as appropriate, stained with a Romanowsky-type stain using a RAL Stainer (CellaVision RAL Diagnostics, Martillac, France) [1].

Effusions were classified according to total protein concentration, cellularity and cytological characteristics. The traditional framework categorised body fluids as transudates, modified transudates or exudates [1,37]. The previously established criteria comprised total protein < 2.5 g/dL and TNCC < 1500 cells/μL for transudates; total protein 2.5–7.5 g/dL and TNCC 1000–7000 cells/μL for modified transudates; and total protein > 3.0 g/dL and TNCC > 7000 cells/μL for exudates [1].

Total protein and albumin concentrations were measured using a Beckman Coulter AU680e analyser (Beckman Coulter, Cassina de’ Pecchi, Italy) by the biuret (Cat. OSR6132, Beckman Coulter^®^, Brea, CA, USA) and bromocresol green methods (Cat. OSR6102, Beckman Coulter^®^, Brea, CA, USA), respectively. Globulin concentration was calculated as total protein minus albumin, and the A/G ratio as albumin divided by globulin. The Rivalta test was performed and interpreted according to the previously described protocol [1,31,33].

Nucleated-cell measurements were obtained using the Sysmex XN-1000V haematology analyser (Sysmex Corporation, Kobe, Japan) in whole-blood mode. ΔTNC was operationally defined as the ratio (1):

(1) $$\Delta TNC=\frac{TNCC\text{-}WDF}{TNCC-WNR}$$

where TNCC-WDF and TNCC-WNR represent the total nucleated cell counts generated by the white blood cell differential and white cell nucleated channels, respectively [1,34,35].

The anatomical site of the sampled effusion was recorded as abdominal, pleural or pericardial. This sample-level classification does not imply that effusion was confined to a single cavity in each cat. Cytological evidence of intracellular bacteria within an inflammatory effusion supported classification as septic, and microbiological culture was considered corroborative when available.

For the prespecified phenotype analysis, modified transudates were compared with effusions belonging to the exudative family. Less frequent phenotypes, comprising chylous, transudative and haemorrhagic effusions, remained included in the overall analysis.

### 2.3. Molecular Analysis and Reference Classification

Viral RNA was extracted from effusion samples using the Promega AX9760 (Promega Corporation, Madison, WI, USA) custom nucleic-acid extraction protocol for whole blood, adapted for body-fluid samples and implemented on the KingFisher™ Flex system (Thermo Fisher Scientific, Waltham, MA, USA), following the previously described laboratory procedure [1]. FCoV RNA was detected using the NZYtech Feline Coronavirus RT-qPCR Kit (Cat. MD05601, NZYtech^®^, Lisbon, Portugal) on a QuantStudio™ 5 Real-Time PCR System (Thermo Fisher Scientific, Waltham, MA, USA), according to the manufacturer’s instructions. Thermal cycling comprised reverse transcription at 50 °C for 20 min, polymerase activation at 95 °C for 2 min, and 40 cycles of denaturation at 95 °C for 5 s and annealing/extension at 60 °C for 30 s [1]. Each reaction included an internal extraction control, and each analytical run included appropriate positive and negative controls. A molecular result was considered analytically valid only when the internal extraction control amplified appropriately, the positive control showed amplification and no target amplification was observed in the negative control. No study-specific quantification-cycle cut-off was introduced. Valid samples were classified according to the manufacturer’s qualitative criteria, with target amplification at a quantification cycle (Cq) < 40 and the appropriate sigmoidal profile considered positive. Valid samples without target amplification were classified as negative. Invalid results were excluded from the diagnostic-accuracy analysis. The qualitative positivity criterion does not distinguish low from high viral loads. Late amplification meeting the same validity and positivity criteria was not assigned a separate study-specific category.

The qualitative effusion FCoV RT-qPCR result constituted the molecular reference classification. Group allocation was based solely on FCoV RT-qPCR and was presumptive with respect to FIP status. Detection of FCoV RNA in effusion is not an error-free reference standard for definitive FIP; diagnostic-performance estimates therefore describe discrimination and classification relative to molecular status, rather than absolute disease status [38,39,40]. Although histopathology with intralesional FCoV antigen detection remains the reference standard, effusion RT-qPCR was selected as the molecular reference classification for this study because effusion sampling is less invasive than tissue biopsy, results are rapidly available and the assay is suitable for routine diagnostic workflows. This choice does not establish independent disease confirmation. The assay detects FCoV without distinguishing feline enteric coronavirus (FECV) from FIP-associated variants (FIPV), and a negative effusion RT-qPCR result alone cannot exclude FIP [30].

### 2.4. FIP Effusion Index and Prespecified Thresholds

The FIP Effusion Index was calculated according to Equation (2) originally proposed by Lopes et al. [1]:

(2) $$FIP\;Effusion\;Index=\frac{\Delta Total\;Nucleated\;Cells\;(\Delta TNC)}{Albumin/Globulin\;(A/G)\;ratio}$$

The FIP Effusion Index was calculated as a continuous variable before application of any diagnostic threshold. The primary index test was evaluated at the prespecified clinical threshold of ≥7.54, representing the rounded form of the ≥7.542 threshold derived in the previous study independently of the present dataset and subsequently cited in the 2026 ABCD guidelines [1,30]. The historical unrounded threshold of ≥7.542 was evaluated separately as a sensitivity analysis and was not used to redefine the primary operating point.

Previously published comparator thresholds were ΔTNC ≥ 4.903, ΔTNC ≥ 2.081 and A/G ≤ 0.56 [1]. Rivalta test positivity was evaluated as a binary comparator.

The primary diagnostic outcomes were sensitivity and specificity. Secondary measures included overall accuracy, positive and negative likelihood ratios (LR+ and LR−), and the diagnostic odds ratio (DOR). Throughout this study, sensitivity, specificity, accuracy, likelihood ratios and true/false-positive or -negative classifications are defined relative to effusion FCoV RT-qPCR, not independently established FIP status.

### 2.5. Statistical Analysis

Continuous variables were summarised as median and interquartile range (IQR), and categorical variables as counts and percentages. Differences between FCoV RT-qPCR-positive and -negative effusions were assessed using two-sided Mann–Whitney U tests, with rank-biserial correlation reported as an effect-size measure.

Diagnostic performance at the prespecified thresholds was evaluated from two-by-two contingency tables. Sensitivity, specificity and accuracy were calculated with exact 95% confidence intervals (CIs), together with LR+, LR− and DORs. Continuous discrimination of the FIP Effusion Index, ΔTNC and A/G ratio was assessed using receiver operating characteristic (ROC) curves and areas under the curve (AUCs). A/G was directionally reversed because lower values support FIP. AUCs were compared using paired DeLong tests [41], with Holm adjustment applied to the three pairwise comparisons.

Fixed-threshold differences in sensitivity and specificity were assessed using exact McNemar tests with Holm adjustment. Prespecified subgroup analyses compared modified transudates with exudates and septic with non-septic effusions; abdominal and pleural effusions were examined exploratorily. Independent proportions were compared using Fisher’s exact test.

The Youden index was used only for exploratory assessment of threshold concordance. AUC and exploratory threshold stability were evaluated using 3000 stratified bootstrap resamples preserving reference-class proportions. No post hoc power calculation was performed. All tests were two-sided, with *p* < 0.05 considered statistically significant [42].

Statistical analyses were performed using JMP^®^ version 18.0.0 (SAS Institute Inc., Cary, NC, USA), DATAtab^®^ (numiqo e.U., Graz, Austria, 2026), MedCalc^®^ Statistical Software version 20.006 (MedCalc Software Ltd., Ostend, Belgium), and R 4.6.1 (R Foundation for Statistical Computing, Vienna, Austria).

## 3. Results

### 3.1. Study Population

The analysis dataset comprised 176 effusion records, of which 92 (52.3%) were FCoV RT-qPCR-positive and 84 (47.7%) were negative. Modified transudates were the predominant phenotype (117/176; 66.5%), followed by exudates (46/176; 26.1%); 13 records (7.4%) comprised seven chylous, five transudative and one haemorrhagic effusion. Septic inflammation was recorded in 23/176 (13.1%) observations. The sampled effusions comprised 118 abdominal (67.0%), 55 pleural (31.3%) and three pericardial (1.7%) effusions. Anatomical location and sex were available for all records. Signalment and principal fluid characteristics are summarised in Table 1.

### 3.2. Continuous Effusion Markers According to Reference Classification

Molecular-reference-positive effusions had higher total protein and globulin concentrations and lower A/G ratios than reference-negative effusions (Table 2). The largest univariable effect sizes were observed for the FIP Effusion Index (rank-biserial *r* = 0.923) and ΔTNC (*r* = 0.894). Median FIP Effusion Index values were 45.90 (IQR 25.41–94.81) in reference-positive effusions and 1.21 (0.86–2.14) in reference-negative effusions (*p* < 0.001; rank-biserial *r* = 0.923). Corresponding median ΔTNC values were 19.09 (9.82–37.00) and 0.98 (0.87–1.17), respectively (both *p* < 0.001). Globulin (*r* = 0.779) and A/G (*r* = −0.775) also showed substantial separation. In contrast, TNCC-WDF alone provided little separation (median 1.987 vs. 2.800 × 10^9^/L; *p* = 0.090; *r* = −0.148). Figure 1 displays the FIP Effusion Index distribution at the locked threshold.

### 3.3. Primary Validation of the Prespecified FIP Effusion Index Threshold

Application of the independently established threshold of ≥7.54, without optimisation in this dataset, classified 90/92 reference-positive and 74/84 reference-negative effusions correctly (TP/FP/FN/TN, 90/10/2/74). Sensitivity was 97.8% (95% CI, 92.4–99.7%), specificity was 88.1% (95% CI, 79.2–94.1%) and overall accuracy was 93.2% (95% CI, 88.4–96.4%). LR+ was 8.22 (95% CI, 4.59–14.71) and LR− was 0.025 (95% CI, 0.006–0.097), corresponding to a DOR of 333 (95% CI, 70.7–1567.4).

Use of the exact historical threshold of ≥7.542 rather than its rounded clinical form resulted in one additional false-negative classification: sensitivity was 96.7%, while specificity remained 88.1%. The clinical threshold of ≥7.54 was therefore retained for all primary and subgroup analyses.

### 3.4. Continuous Discrimination and Incremental Value

The continuous FIP Effusion Index showed excellent discrimination, with an AUC of 0.962 (95% CI, 0.928–0.995). The AUCs of ΔTNC and A/G were 0.947 (95% CI, 0.910–0.984) and 0.887 (95% CI, 0.837–0.937), respectively (Figure 2). Because ROC AUC evaluates the continuous marker, the ΔTNC AUC is independent of the fixed cut-offs. The operating characteristics at ΔTNC ≥ 4.903 and ≥2.081 are reported separately in Table 3. The complementary two-dimensional relationship between ΔTNC and A/G at the fixed FIP Effusion Index threshold is shown in Figure 3. In paired DeLong analysis, the FIP Effusion Index exceeded ΔTNC by 0.014 AUC units (95% CI, 0.004–0.025; *p* < 0.01; Holm-adjusted *p* = 0.012) and A/G by 0.074 (95% CI, 0.024–0.125; *p* < 0.01; adjusted *p* = 0.012). ΔTNC also exceeded A/G (ΔAUC, 0.060; 95% CI, 0.004–0.115; *p* = 0.034; adjusted *p* = 0.034). Differences were calculated from unrounded AUC estimates. Thus, combining ΔTNC with A/G produced a statistically demonstrable, although quantitatively modest, improvement over ΔTNC alone.

### 3.5. Paired Fixed-Threshold Comparisons

At fixed cut-offs, the FIP Effusion Index correctly identified six reference-positive effusions missed by ΔTNC ≥ 4.903, while no reference-positive effusion was correctly identified by ΔTNC and missed by the Index. The exact McNemar *p* value was 0.031. However, this difference did not remain significant after Holm adjustment across the prespecified fixed-threshold comparison family (adjusted *p* = 0.125). In contrast, the sensitivity advantage of the FIP Effusion Index over A/G ≤ 0.56 remained significant (adjusted *p* < 0.01). The principal fixed-threshold advantage over the Rivalta test was specificity: among reference-negative records, 17 were correctly classified by the FIP Effusion Index but positive by the Rivalta test, with no discordance in the opposite direction (exact *p* < 0.001; adjusted *p* < 0.001).

### 3.6. Performance Beyond Modified Transudates

The prespecified threshold retained high sensitivity in both major effusion phenotypes. Among 117 modified transudates, sensitivity was 97.3% (95% CI, 90.7–99.7%) and specificity was 92.9% (95% CI, 80.5–98.5%), with an AUC of 0.970. Among 46 exudates, all 17 reference-positive records were FIP Effusion Index-positive, giving an observed sensitivity of 100% (95% CI, 80.5–100%); specificity was 82.8% (95% CI, 64.2–94.2%) and the AUC was 0.947 (95% CI, 0.876–1.000). Sensitivity did not differ between modified transudates and exudates (Fisher *p* = 1.000). Specificity was 10.1 percentage points higher in modified transudates than in exudates (difference, +10.1 percentage points; 95% CI, −5.2–28.0; Fisher *p* = 0.258). These results provide no evidence that exudative classification causes loss of sensitivity. Within the exudative samples, no misclassification occurred among 26 non-septic effusions (14 reference-positive and 12 reference-negative), whereas 5/17 reference-negative septic exudates were FIP Effusion Index-positive. The septic-exudate subgroup nevertheless retained an AUC of 0.941, suggesting that the continuous marker remained strongly informative even though a subset of severe inflammatory effusions crossed the fixed threshold. Given the small subgroup sizes and zero cells in some strata, these estimates should be interpreted through their confidence intervals rather than as evidence of perfect subgroup classification.

### 3.7. Septic Inflammation and False-Positive Classification

Septic inflammation emerged as the principal identifiable context associated with reduced specificity. Across all molecular reference-negative effusions, false-positive FIP Effusion Index results occurred in 6/19 (31.6%) septic records compared with 4/65 (6.2%) non-septic records. Specificity was therefore 68.4% in septic effusions and 93.8% in non-septic effusions, corresponding to a difference of −25.4 percentage points (95% CI, −48.1 to −7.1; Fisher *p* < 0.01). The odds of false-positive classification were approximately seven-fold higher in septic than in non-septic reference-negative effusions (OR, 7.04; 95% CI, 1.74–28.53). The distribution of observations according to molecular reference and septic status is shown in Figure 3.

Notably, septic inflammation did not invariably result in an increased FIP Effusion Index (Table 4). A representative FCoV RT-qPCR-negative septic exudate correctly classified below the prespecified threshold is shown in Figure 4 and Figure 5. Despite marked inflammatory cytological findings, this effusion had a ΔTNC of 1.00, an A/G ratio of 1.19 and a resulting FIP Effusion Index of 0.84. This example illustrates that even marked septic suppurative inflammation does not necessarily reproduce the inter-channel discordance associated with a high FIP Effusion Index.

### 3.8. Exploratory Threshold Stability and Bootstrap Analysis

Exploratory Youden analysis in the full validation cohort showed that the prespecified threshold of 7.54 achieved the maximum Youden J (0.859), with a sensitivity of 97.8% and a specificity of 88.1%. The algorithm-derived optimal threshold was 6.300, which yielded identical sensitivity, specificity, and Youden J to the prespecified threshold. Across 3000 stratified bootstrap resamples, the median algorithm-derived optimal threshold was 6.300 (IQR, 6.171–6.362), with a broad 2.5th–97.5th percentile range of 3.62–17.42, indicating appreciable finite-sample instability in data-driven cut-off selection. The bootstrap AUC distribution was more stable (median, 0.964; percentile range, 0.924–0.990). These exploratory findings show that the prespecified threshold achieves the maximum Youden performance in the validation cohort, while the bootstrap results illustrate the instability of selecting a single data-driven optimal threshold; they do not retroactively validate or re-derive the prespecified cut-off (Figure 6).

## 4. Discussion

This study was designed not to redefine the cut-off, but to determine whether the previously established FIP Effusion Index and its prespecified threshold [1] remained informative in a temporally distinct, substantially larger cohort spanning a broader spectrum of effusions. Four clinically important findings emerged. First, the threshold of ≥7.54 retained very high sensitivity (97.8%; 95% CI, 92.4–99.7%), strong overall discrimination (AUC, 0.962; 95% CI, 0.928–0.995) and a DOR of 333 (95% CI, 70.7–1567) in a cohort more than three times larger than the original derivation series [1]. Second, the composite FIP Effusion Index discriminated reference-positive from reference-negative effusions better than either ΔTNC or A/G alone, although the gain over ΔTNC was quantitatively modest. Third, high sensitivity was retained among exudates, indicating that an exudative phenotype did not abolish the analytical signal captured by the Index. Fourth, septic inflammation was the most evident context associated with reduced specificity. These observations define both the potential clinical value and a clinically recognisable limitation of the FIP Effusion Index without equating molecular-reference positivity with independently confirmed FIP.

The validation strategy is a major strength of the present work. Both the FIP Effusion Index formula and the clinical threshold were established in an independent derivation cohort and were locked before the present data were analysed [1]. This distinction matters because post hoc threshold selection can produce optimistic estimates that fail to generalise [43,44]. Temporal separation, inclusion of all eligible cats during the predefined period and deliberate retention of inflammatory competitors therefore constitute a more stringent test of transportability. Exploratory Youden analysis showed that the locked threshold of 7.54, subsequently reproduced in ABCD guidelines [30], achieved the same maximum Youden J as the algorithm-derived optimal threshold of 6.300, with identical sensitivity and specificity, thereby supporting concordance with the previously established threshold rather than its retrospective re-derivation. The median bootstrap algorithm-derived optimal threshold was 6.300, with a broad percentile range (3.62–17.42), further illustrating the instability of data-driven cut-off selection in finite validation samples and why an established clinical threshold should not be repeatedly recalibrated.

The present findings extend, rather than simply reproduce, the original FIP Effusion Index study [1] and the earlier ΔTNC literature [10,34,35]. In the 50-effusion derivation cohort, the ≥7.542 threshold yielded 96.3% sensitivity and 100% specificity [1]. In this study, sensitivity was similar, but specificity was lower at 88.1% (95% CI, 79.2–94.1%). These point estimates should not be interpreted as directly interchangeable because the present sample was larger and included septic, exudative and otherwise diagnostically challenging fluids that had been restricted or excluded during derivation. Earlier XT-2000iV studies established ΔTNC as a highly discriminating effusion marker [10,34,35], whereas subsequent comparison of the XT-2000iV and XN-1000V demonstrated systematic inter-analyser differences despite broadly similar diagnostic classification [11]. Thus, the current results strengthen the evidence for the FIP Effusion Index specifically on the XN-1000V under the stated pre-analytical and analytical conditions. Consequently, neither ΔTNC values nor the FIP Effusion Index threshold should be assumed to be transferable to another analyser without platform-specific validation.

The paired analyses help explain why integration is useful. ΔTNC, operationally the ratio of TNCC-WDF to TNCC-WNR, captures inter-channel discordance arising from distinct reagent and measurement environments, whereas A/G describes the distribution of albumin relative to globulins within the inflammatory protein milieu. Protein-rich FIP effusions, monocyte/macrophage-driven inflammation and altered vascular permeability are well documented [45,46,47], and fibrin- or protein-associated cellular aggregation or trapping in one analytical channel has been proposed as a contributor to ΔTNC [10,11,34]. The precise physicochemical mechanism on the XN-1000V has not been experimentally resolved, however, and this explanation should remain a hypothesis rather than be presented as established analyser behaviour. By combining partially complementary cellular/analytical and biochemical information, the FIP Effusion Index increased AUC by 0.014 over ΔTNC (95% CI, 0.004–0.025; Holm-adjusted *p* = 0.012) and by 0.074 over A/G (95% CI, 0.024–0.125; adjusted *p* = 0.012). The gain over ΔTNC was statistically supported but quantitatively modest. At fixed thresholds, the FIP Effusion Index recovered six reference-positive effusions missed by ΔTNC ≥ 4.903, but this sensitivity comparison did not remain significant after multiplicity adjustment (adjusted *p* = 0.125). The broader principle that ratios integrating distinct effusion measurements may improve diagnostic discrimination is also supported by previous work evaluating the effusion lactate dehydrogenase (LDH)/TNCC ratio, although that marker and the present FIP Effusion Index are not analytically or clinically interchangeable [48].

Diagnostic performance in exudates is a central advance of this validation. Effusive FIP is conventionally associated with a protein-rich, low-to-moderate-cellularity modified transudate, but both current guidelines and series of feline effusions demonstrate substantial overlap between cytological categories and aetiologies [12,30,49,50,51]. Traditional transudate, modified-transudate and exudate labels describe fluid characteristics; they are neither diagnostic nor disease-specific. All 17 reference-positive exudates were FIP Effusion Index-positive, corresponding to an observed sensitivity of 100%, but the 95% CI of 80.5–100% and the small denominator preclude any claim of perfect performance. Specificity in exudates was 82.8% (95% CI, 64.2–94.2%), and the data provided no evidence that sensitivity differed from that in modified transudates. Accordingly, exudative classification alone should neither exclude FIP from the differential diagnosis nor invalidate interpretation of the Index. Conversely, an exudative FIP Effusion Index-positive effusion should not be presumed to represent FIP, particularly when cytological evidence of bacterial inflammation is present.

The lower overall specificity compared with derivation [1] is therefore scientifically informative. Diagnostic performance varies with case mix, competing disorders and the distribution of disease severity, even when the test itself is unchanged [44,52]. In this cohort, specificity was 93.8% (95% CI, 85.0–98.3%) among non-septic effusions but 68.4% (95% CI, 43.5–87.4%) among septic effusions. The broader cohort is consequently a harder and more clinically relevant challenge than the restricted derivation set, but it should not be assumed to represent every intended-use population. The results locate much of the observed loss of specificity within a defined inflammatory context rather than supporting a general loss of discrimination by the FIP Effusion Index.

Among the 84 reference-negative effusions, false-positive classification occurred in 6/19 (31.6%) septic samples and 4/65 (6.2%) non-septic samples, an absolute specificity difference of −25.4 percentage points (95% CI, −48.1 to −7.1) and an unadjusted odds ratio of 7.04 (95% CI, 1.74–28.53). This establishes an association, not causation or independence from effusion phenotype. The analysis was univariable, and septic status was concentrated among exudates. Overlap in globulin enrichment, low A/G, fibrin content and cellular–reagent behaviour offers plausible explanations, but these mechanisms were not directly measured [40,45,46,53,54]. Equally, sepsis did not invariably produce a high FIP Effusion Index: the representative septic exudate had marked suppurative inflammation but a ΔTNC of 1.00, A/G of 1.19 and FIP Effusion Index of 0.84. Severe cytological inflammation alone is therefore insufficient to reproduce the complete signature. Clinically, an FIP Effusion Index-positive effusion containing degenerate neutrophils and intracellular bacteria warrants increased caution and integration with cytology, bacterial culture where available, direct FCoV testing and the full clinical context [30,55]. Septic effusions should not simply be excluded from interpretation, and bacterial inflammation should not automatically be treated as mutually exclusive with FIP because secondary bacterial complications can occur [30].

Likelihood ratios translate these operating characteristics more usefully than sensitivity and specificity alone [56]. The LR− of 0.025 (95% CI, 0.006–0.097) was particularly strong in this cohort. For an assumed 50% pre-test probability of effusion RT-qPCR positivity, the pre-test odds are 1.0. Multiplying by 0.025 gives post-test odds of 0.025 and a post-test probability of approximately 2.4%. A negative FIP Effusion Index result would therefore markedly reduce the probability of molecular-reference positivity under that assumption. By contrast, at an assumed pre-test probability of 10%, the LR+ of 8.22 changes odds from 0.111 to 0.913, equivalent to a post-test probability of approximately 47.7%: a substantial increase, but not a definitive result. These examples concern the molecular reference used here, not histopathologically confirmed FIP.

The FIP Effusion Index should also be positioned alongside, rather than in opposition to, conventional tests. The Rivalta test and A/G remain inexpensive, rapid and widely accessible, and neither requires the specified haematology platform [2,3,30,55]. In the present paired data, the Rivalta test retained high sensitivity (96.7%) but lower specificity (67.9%). The FIP Effusion Index correctly classified 17 reference-negative effusions that were Rivalta-positive, with no discordance in the opposite direction. Low A/G is common in confirmed FIP but also accompanies other inflammatory states, limiting its stand-alone specificity [17,45,55]. Where TNCC-WDF, TNCC-WNR and effusion biochemistry are already available, their integration therefore provides greater discrimination than either component alone and may improve specificity over the Rivalta test. Direct FCoV antigen assays on effusion macrophages provide different information but have also shown variable operating characteristics across studies [29,57,58,59]. A recent diagnostic clinical trial of multiplex fluorescent immunocytochemistry likewise concluded that the assay was complementary rather than definitive [59]. Collectively, these data favour a multimodal diagnostic workflow over replacement of clinical reasoning by any single effusion test.

The choice of reference classification sets an essential boundary around the present inferences. Effusion FCoV RT-qPCR is clinically valuable because viral RNA is often abundant in FIP-associated effusions [60]. Previous studies reported sensitivities of 85.0% and 88.9% and specificities of 100% relative to their respective disease classifications [20,21]. Nevertheless, RT-qPCR detects FCoV RNA rather than the defining tissue lesion and is not a perfect reference standard [20,21,24,30,55]. Immunocytochemical and immunofluorescent detection of viral antigen in effusion macrophages is also imperfect [29,57,58,59]. The 2026 ABCD guidance identified two unresolved evidence needs for the FIP Effusion Index: evaluation in a larger sample and evaluation in cats with confirmed FIP [30]. This study substantially addresses the first through temporal validation in 176 cats, broader fluid phenotypes, inclusion of septic and exudative competitors and application of the original locked threshold. It does not fully address the second because classification was not based on an independently adjudicated disease diagnosis. The next step should therefore move beyond further analytical validation towards prospective multicentre disease-level evaluation of the FIP Effusion Index within a sequential multivariable diagnostic framework, in which its high sensitivity supports initial rule-out of FIP, while positive cases undergo more specific confirmatory testing, particularly FCoV antigen detection by immunocytochemistry in effusion-derived cell blocks and/or immunohistochemistry in tissue lesions. Such an approach could maximise sensitivity at the screening stage and specificity at the confirmatory stage and could thereby improve the reliability of in vivo FIP diagnosis.

### 4.1. Limitations

Several limitations require explicit consideration. Most importantly, the reference classification was effusion FCoV RT-qPCR rather than histopathology with intralesional FCoV antigen demonstration. Although effusion RT-qPCR is highly informative in the appropriate clinical context, it is not a perfect reference standard for definitive FIP diagnosis [2,3,20,30,38,39]. Consequently, this study addresses the guideline request for a larger and broader validation cohort but does not fully satisfy the separate requirement for a large series of independently confirmed FIP cases [30]. The retrospective, single-centre design may be subject to referral and spectrum effects, and performance is platform- and protocol-dependent. Also, the retrospective nature of the design limited control over case selection, completeness of ancillary diagnostic information and standardisation of clinical follow-up. Although all 176 samples represented unique cats, final alternative diagnoses, standardised case-level descriptions of the two false-negative effusions and complete bacteriological data were not consistently available. One lymphomatous effusion was included, but the available records did not permit systematic assessment of lymphoma or other non-septic disorders as independent sources of false-positive results. The sepsis analysis was univariable, septic status was concentrated among exudates and subgroup precision was limited for septic reference-positive effusions and less frequent fluid categories. In addition, septic effusions were deliberately retained to challenge specificity under clinically realistic inflammatory conditions, although the number of septic samples remained modest, particularly among molecular-reference-positive cats. Therefore, subgroup estimates for septic effusions should be interpreted cautiously and primarily as hypothesis-generating with respect to the magnitude of specificity loss.

### 4.2. Recommendations

From a practical diagnostic perspective, the FIP Effusion Index may be most appropriately positioned as an early effusion-based triage test rather than as a stand-alone definitive diagnostic test. In cats with compatible clinical signs and cavitary effusion, routine cytology, total protein, A/G ratio and automated cell-count data could be integrated at an initial diagnostic stage. A negative FIP Effusion Index would substantially reduce suspicion of FIP and may support prioritisation of alternative diagnoses, particularly when cytology and clinical findings are discordant with FIP. Conversely, a positive FIP Effusion Index should increase diagnostic suspicion and prompt direct FCoV testing. In particular, FCoV antigen detection by immunocytochemistry on effusion-derived cell preparations or cell blocks and/or immunohistochemistry on tissue lesions, when tissue is available, should be used as more specific confirmatory evidence.

Future studies should evaluate the FIP Effusion Index within a disease-level diagnostic framework using FCoV antigen detection by immunohistochemistry in tissue lesions and/or immunocytochemistry in cell-block preparations obtained from effusion samples. Prospective multicentre validation should use the locked threshold of 7.54 or higher on the Sysmex XN-1000V, blinded interpretation and an independently adjudicated reference incorporating histopathology, FCoV antigen detection and clinical follow-up. Challenging inflammatory conditions should be retained, with systematic bacterial culture and, where available, matrix-assisted laser desorption/ionisation time-of-flight mass spectrometry to investigate whether different bacterial aetiologies produce distinct cellular or protein responses and false-positive risks. Reproducibility across analysers and sites should also be assessed. A further priority is the development and external validation of this sequential diagnostic approach, ideally incorporating the FIP Effusion Index within a multivariable clinical–laboratory score. Within such an approach, the highly sensitive FIP Effusion Index could primarily support FIP rule-out when negative, whereas Index-positive cases would undergo more specific confirmatory testing. This strategy could maximise sensitivity at the screening stage and specificity at the confirmatory stage and could thereby improve the reliability of in vivo FIP diagnosis.

## 5. Conclusions

The prespecified FIP Effusion Index threshold of ≥7.54 was validated against effusion FCoV RT-qPCR in a subsequent, substantially larger and diagnostically broader cohort. The FIP Effusion Index retained very high sensitivity across modified-transudative and exudative phenotypes and showed greater continuous discrimination than either ΔTNC or A/G alone, although the gain over ΔTNC was modest. Septic inflammation, rather than exudative phenotype per se, was the principal identifiable limitation to specificity. Accordingly, a negative FIP Effusion Index provides strong evidence against molecular-reference positivity, whereas a positive result, particularly in a septic effusion, should be interpreted alongside cytology, bacterial culture and direct FCoV testing. These results support the FIP Effusion Index as a robust adjunctive effusion biomarker on the Sysmex XN-1000V.

## Figures and Tables

**Figure 1:**
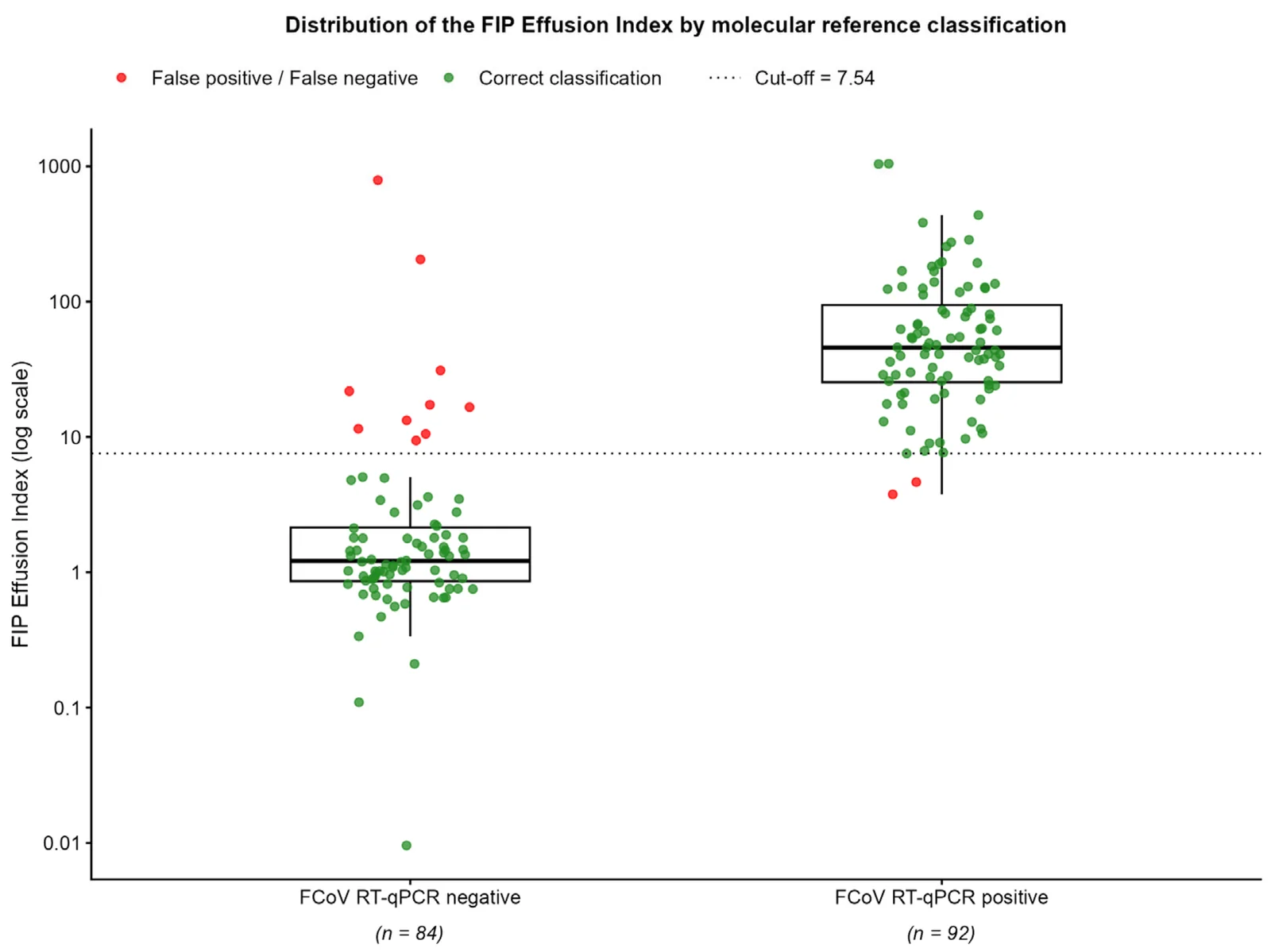
Distribution of the FIP Effusion Index according to the effusion FCoV RT-qPCR molecular reference classification. Individual observations are displayed over box plots on a logarithmic scale. The dashed horizontal line denotes the prespecified clinical cut-off of 7.54. Red symbols identify observations misclassified at this threshold; green symbols identify correctly classified observations. Boxes span the interquartile range (IQR), and central lines denote medians. FCoV, feline coronavirus; RT-qPCR, reverse-transcription quantitative polymerase chain reaction.

**Figure 2:**
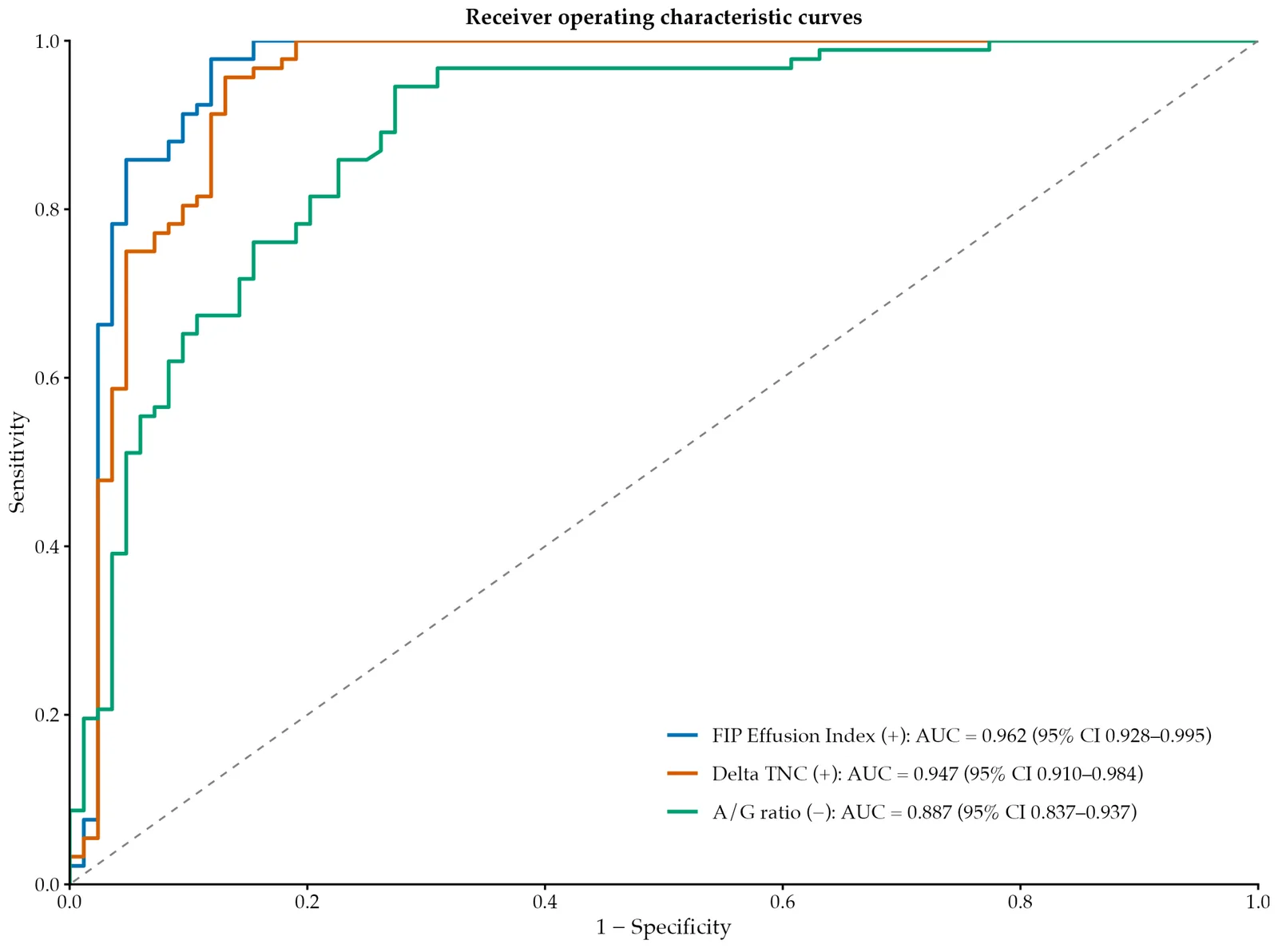
Receiver operating characteristic (ROC) curves for the FIP Effusion Index, ΔTNC and A/G ratio against the effusion FCoV RT-qPCR reference classification. A/G was directionally inverted for ROC analysis because lower values support FIP. The dashed diagonal line represents chance discrimination (AUC = 0.5). A/G, albumin-to-globulin ratio; AUC, area under the ROC curve; CI, confidence interval; FCoV, feline coronavirus; RT-qPCR, reverse-transcription quantitative polymerase chain reaction; ΔTNC, delta total nucleated cells.

**Figure 3:**
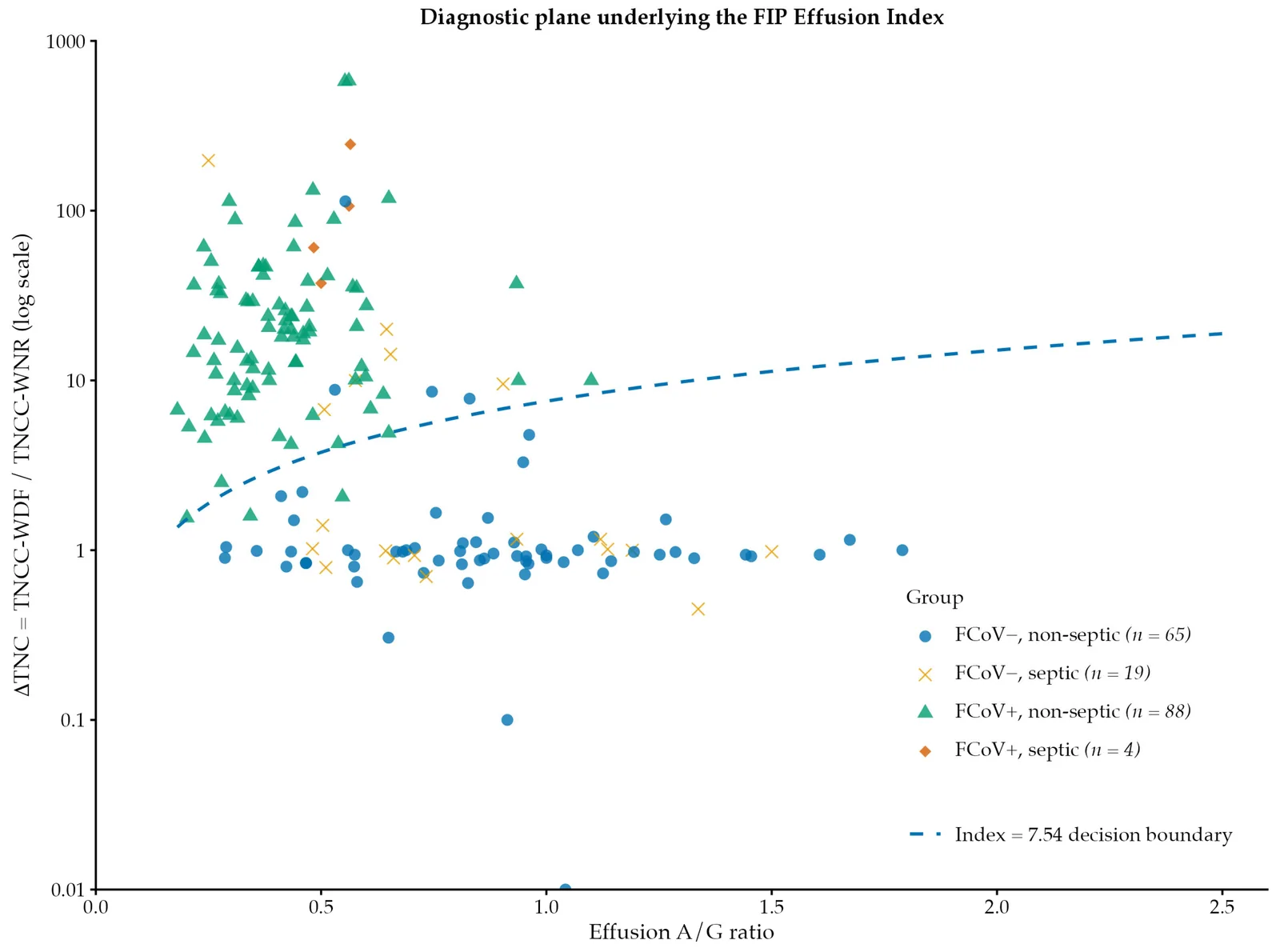
Diagnostic plane underlying the FIP Effusion Index. Each observation is positioned according to its effusion A/G ratio and ΔTNC, with marker shape distinguishing molecular-reference and septic status. The dashed threshold line corresponds to ΔTNC = 7.54 × A/G. Observations above this line have a FIP Effusion Index ≥ 7.54. The logarithmic ΔTNC axis permits simultaneous visualisation of low and markedly increased inter-channel ratios. For visualisation, the x-axis was truncated at an A/G ratio of 2.5. One extreme observation (A/G = 19.0) falls outside the displayed range. A/G, albumin-to-globulin ratio; ΔTNC, delta total nucleated cells.

**Figure 4:**
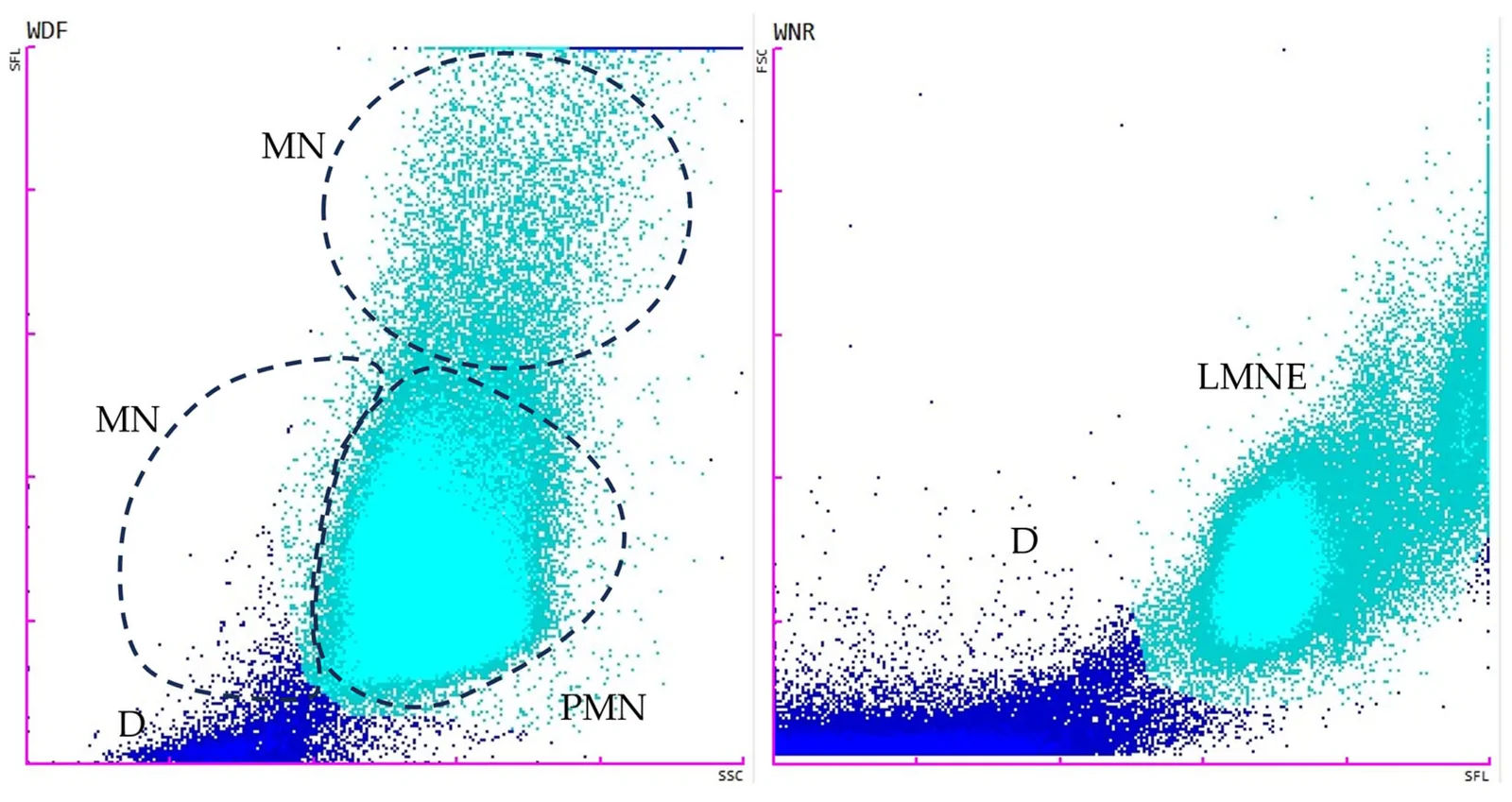
Representative Sysmex XN-1000V scattergrams from a septic exudate analysed in whole-blood mode. The (**left**) panel shows the white blood cell differential (WDF) channel, from which TNCC-WDF was obtained, whereas the (**right**) panel shows the white cell nucleated (WNR) channel, from which TNCC-WNR was obtained. Dashed ellipses outline the regions annotated as MN and PMN in the WDF scattergram and are provided as visual guides rather than validated classification gates. D (dark blue), debris; LMNE (light blue), leucocyte populations other than basophils; MN, mononuclear cells, including macrophages, monocytes and lymphocytes; PMN, polymorphonuclear cells, predominantly neutrophils. In this septic exudate, the relative preservation of nucleated-cell events between the WDF and WNR channels resulted in a low inter-channel ratio (ΔTNC = 1.00). TNCC, total nucleated cell count; ΔTNC, delta total nucleated cells.

**Figure 5:**
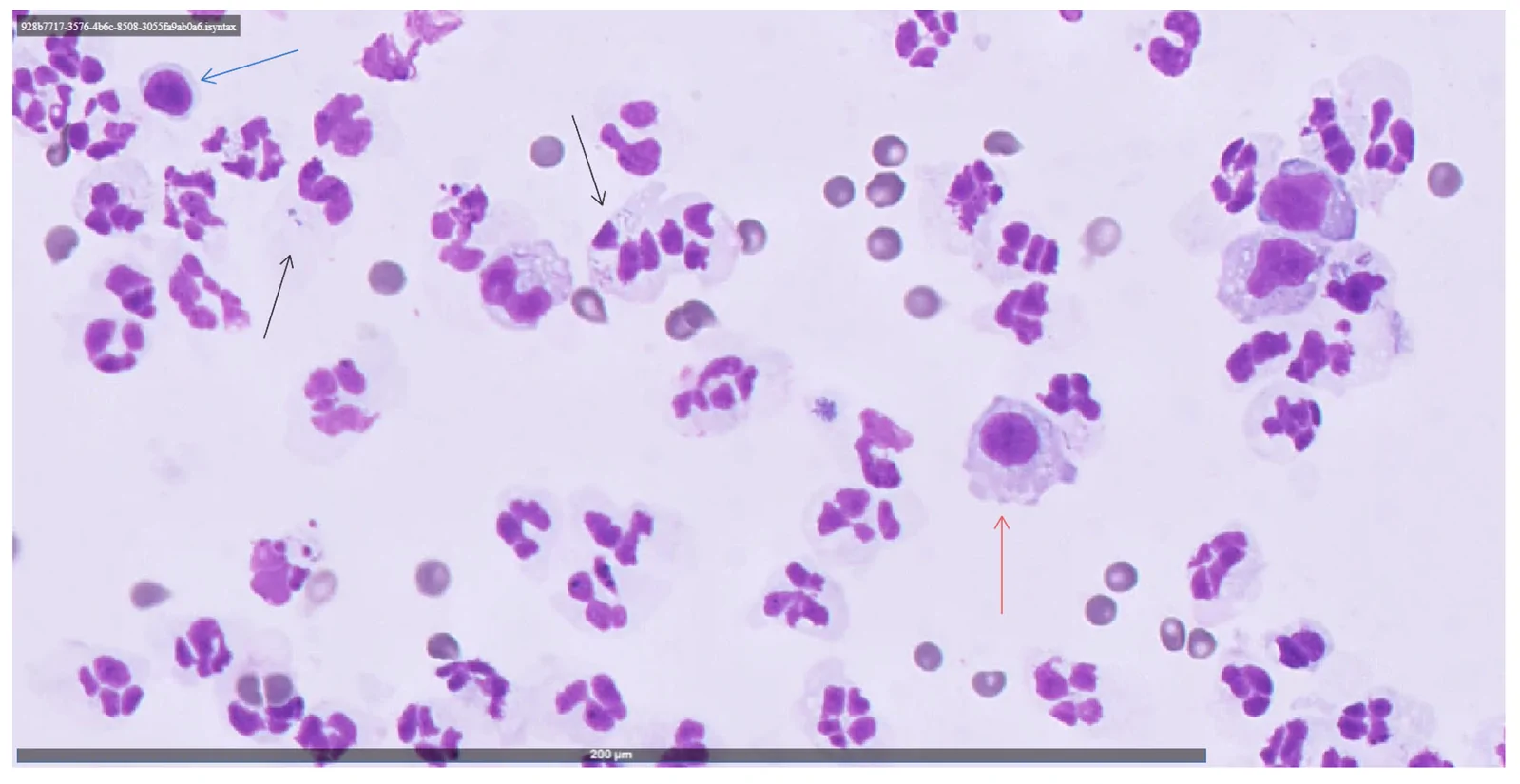
Corresponding cytological appearance of the septic exudate analysed on the Sysmex XN-1000V in whole-blood mode. May–Grünwald–Giemsa-stained preparation showing marked suppurative inflammation characterised by numerous predominantly degenerate neutrophils. Black arrows indicate bacteria within degenerate neutrophils; the red arrow indicates a macrophage; and the blue arrow indicates a reactive lymphocyte. Scattered erythrocytes are present in the background. The slide was digitised using the Philips IntelliSite Pathology Solution, version 3.2 (Philips, Best, The Netherlands), and subsequently visualised using Concentriq^®^ AP-Dx v4.12 (Proscia Inc., Philadelphia, PA, USA). Scale bar = 200 µm.

**Figure 6:**
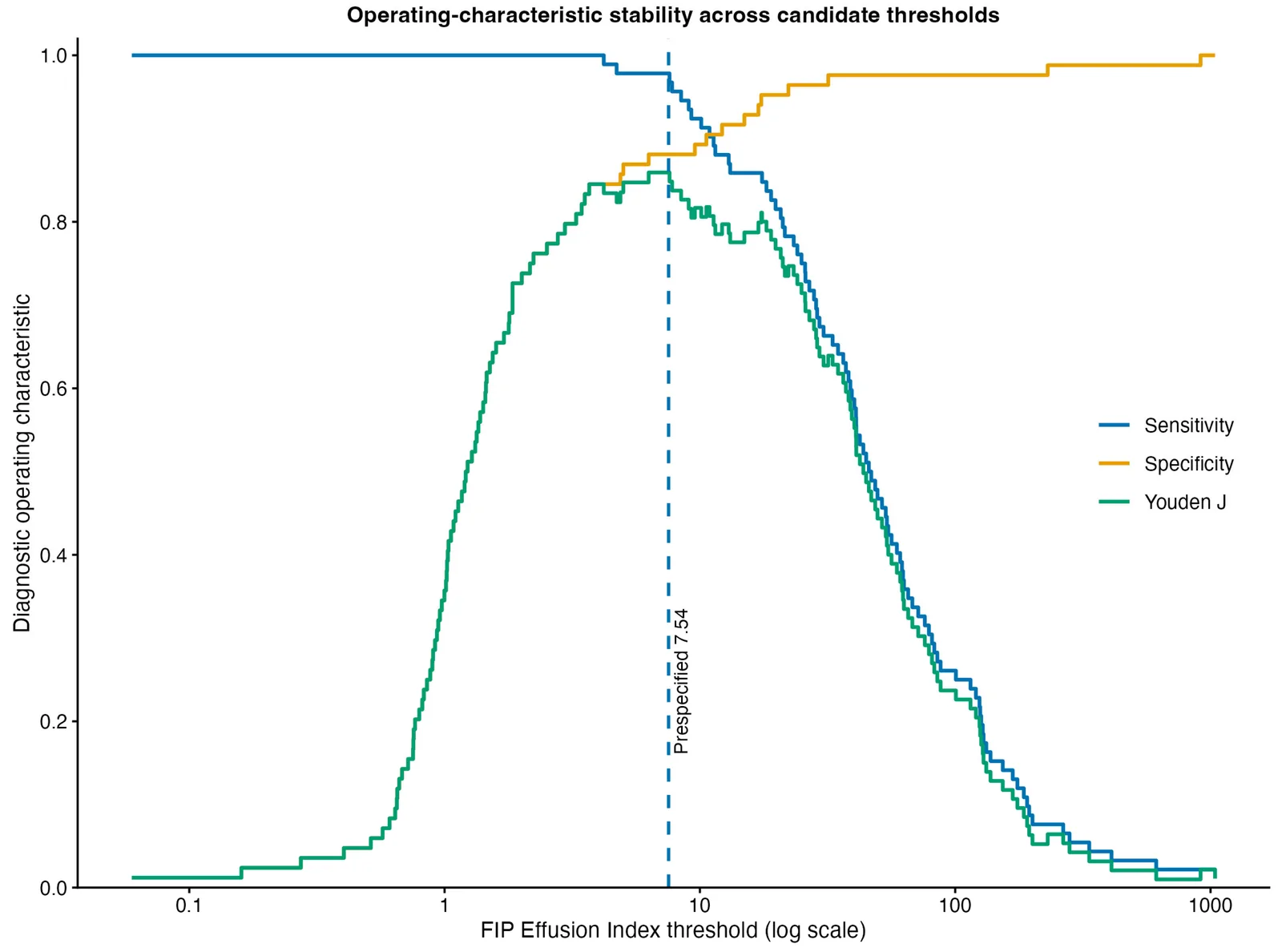
Sensitivity, specificity and Youden J across candidate FIP Effusion Index thresholds. The x-axis is logarithmic to display the broad observed range. The dashed vertical line denotes the prespecified cut-off of 7.54, which achieved the maximum Youden J in the validation cohort. This analysis was used to assess threshold concordance and not to redefine the primary cut-off.

**Table 1:** Demographic, anatomical and cytological characteristics of the expanded-spectrum validation cohort.

Characteristic	Overall	FCoV RT-qPCR Positive	FCoV RT-qPCR Negative
Records, *n*	176	92	84
Age, years, median [IQR]	2.5 [0.8–7.0]	1.0 [0.5–3.0]	6.0 [2.0–10.0]
Female sex, *n* (%)	82 (46.6)	44 (47.8)	38 (45.2)
Male sex, *n* (%)	94 (53.4)	48 (52.2)	46 (54.8)
Abdominal effusion, *n* (%)	118 (67.0)	73 (79.3)	45 (53.6)
Pleural effusion, *n* (%)	55 (31.3)	19 (20.7)	36 (42.9)
Pericardial effusion, *n* (%)	3 (1.7)	0 (0.0)	3 (3.6)
Modified transudate, *n* (%)	117 (66.5)	75 (81.5)	42 (50.0)
Exudate, *n* (%)	46 (26.1)	17 (18.5)	29 (34.5)
Other phenotypes, *n* (%)	13 (7.4)	0 (0.0)	13 (15.5)
Septic, *n* (%)	23 (13.1)	4 (4.3)	19 (22.6)
Rivalta test positive, *n* (%)	116 (65.9)	89 (96.7)	27 (32.1)

Age was available for 154/176 records (83 reference-positive; 71 reference-negative). Percentages are column percentages. Sex and anatomical location were available for all records. The exudative family comprised 44 inflammatory exudates, one carcinomatous/septic exudate and one lymphomatous exudate; ‘other’ comprised seven chylous, five transudative and one haemorrhagic effusion.

**Table 2:** Distribution of continuous diagnostic variables according to FCoV RT-qPCR reference classification.

Variable	Reference Positive Median [IQR]	Reference Negative Median [IQR]	*p* Value	Rank-Biserial *r*
Total protein (g/dL)	5.76 [5.13–6.72]	4.08 [3.32–5.01]	<0.001	0.671
Albumin (g/dL)	1.65 [1.46–1.86]	1.90 [1.50–2.17]	0.0026	−0.264
Globulins (g/dL)	4.11 [3.50–4.99]	2.09 [1.77–2.89]	<0.001	0.779
A/G ratio	0.407 [0.309–0.488]	0.828 [0.576–1.039]	<0.001	−0.775
TNCC-WDF (×10^9^/L)	1.987 [0.668–6.062]	2.800 [0.718–17.661]	0.090	−0.148
ΔTNC	19.09 [9.82–37.00]	0.98 [0.87–1.17]	<0.001	0.894
FIP Effusion Index	45.90 [25.41–94.81]	1.21 [0.86–2.14]	<0.001	0.923

*p* values are from two-sided Mann–Whitney U tests. The sign of the rank-biserial coefficient denotes the direction of higher values in the reference-positive group. IQR, interquartile range; FCoV, feline coronavirus; RT-qPCR, reverse-transcription quantitative polymerase chain reaction; A/G, albumin-to-globulin ratio; TNCC-WDF and TNCC-WNR, total nucleated cell counts generated by the white blood cell differential and white cell nucleated channels, respectively; ΔTNC, TNCC-WDF/TNCC-WNR ratio.

**Table 3:** Diagnostic performance of the prespecified FIP Effusion Index and published comparator thresholds against effusion FCoV RT-qPCR.

Test/Threshold	TP/FP/FN/TN	Sensitivity (%) (95% CI)	Specificity (%) (95% CI)	DOR (95% CI)	LR+	LR−	Accuracy (%)
FIP Effusion Index ≥ 7.54	90/10/2/74	97.8 (92.4–99.7)	88.1 (79.2–94.1)	333.0 (70.7–1567.4)	8.22	0.025	93.2
FIP Effusion Index ≥ 7.542	89/10/3/74	96.7 (90.8–99.3)	88.1 (79.2–94.1)	219.5 (58.3–827.2)	8.13	0.037	92.6
ΔTNC ≥ 4.903	84/10/8/74	91.3 (83.6–96.2)	88.1 (79.2–94.1)	77.7 (29.1–207.2)	7.67	0.099	89.8
ΔTNC ≥ 2.081	89/14/3/70	96.7 (90.8–99.3)	83.3 (73.6–90.6)	148.3 (41.0–536.5)	5.80	0.039	90.3
A/G ≤ 0.56	75/19/17/65	81.5 (72.1–88.9)	77.4 (67.0–85.8)	15.1 (7.2–31.4)	3.61	0.239	79.5
Rivalta positive	89/27/3/57	96.7 (90.8–99.3)	67.9 (56.8–77.6)	62.6 (18.2–216.1)	3.01	0.048	83.0

Sensitivity and specificity are shown with exact 95% CIs. DORs are presented with log-normal 95% CIs. CI, confidence interval; TP, true positive; FP, false positive; FN, false negative; TN, true negative; LR+, positive likelihood ratio; LR−, negative likelihood ratio; DOR, diagnostic odds ratio.

**Table 4:** Performance of the FIP Effusion Index ≥ 7.54 across prespecified clinical subgroups.

Subgroup	*n*	TP/FP/FN/TN	Sensitivity (%) (95% CI)	Specificity (%) (95% CI)	AUC (95% CI)
Overall	176	90/10/2/74	97.8 (92.4–99.7)	88.1 (79.2–94.1)	0.962 (0.928–0.995)
Modified transudate	117	73/3/2/39	97.3 (90.7–99.7)	92.9 (80.5–98.5)	0.970 (0.927–1.000)
Exudate	46	17/5/0/24	100 (80.5–100)	82.8 (64.2–94.2)	0.947 (0.876–1.000)
Non-septic exudate	26	14/0/0/12	100 (76.8–100)	100 (73.5–100)	1.000 (CI not estimable)
Septic exudate	20	3/5/0/12	100 (29.2–100)	70.6 (44.0–89.7)	0.941 (0.826–1.000)
All non-septic	153	86/4/2/61	97.7 (92.0–99.7)	93.8 (85.0–98.3)	0.979 (0.950–1.000)
All septic	23	4/6/0/13	100 (39.8–100)	68.4 (43.5–87.4)	0.947 (0.844–1.000)
Abdominal	118	71/8/2/37	97.3 (90.5–99.7)	82.2 (67.9–92.0)	0.936 (0.875–0.996)
Pleural	55	19/2/0/34	100 (82.4–100)	94.4 (81.3–99.3)	0.991 (0.976–1.000)

Observed values of 100% are accompanied by exact confidence intervals (CI) where estimable and should not be interpreted as proof of perfect diagnostic performance. Abdominal and pleural analyses were exploratory secondary analyses. Pericardial effusions (*n* = 3, all reference-negative) were not analysed separately because sensitivity and AUC were not estimable.

## Data Availability

The data presented in this study are available upon request from the corresponding authors.

## Abbreviations

The following abbreviations are used in this manuscript:

A/G	albumin-to-globulin ratio
ABCD	European Advisory Board on Cat Diseases
AUC	area under the receiver operating characteristic curve
CI	confidence interval
DOR	diagnostic odds ratio
EDTA	ethylenediaminetetraacetic acid
FCoV	feline coronavirus
FIP	feline infectious peritonitis
FN	false negative
FP	false positive
IHC	immunohistochemistry
IQR	interquartile range
LDH	lactate dehydrogenase
LR+	positive likelihood ratio
LR−	negative likelihood ratio
OR	odds ratio
RNA	ribonucleic acid
ROC	receiver operating characteristic
RT-qPCR	reverse-transcription quantitative polymerase chain reaction
STARD	Standards for Reporting of Diagnostic Accuracy
TN	true negative
TNCC	total nucleated cell count
TNCC-WDF	total nucleated cell count generated by the white blood cell differential channel
TNCC-WNR	total nucleated cell count generated by the white cell nucleated channel
TP	true positive
WDF	white blood cell differential
WNR	white cell nucleated
ΔAUC	difference in area under the receiver operating characteristic curve
ΔTNC	delta total nucleated cells
